# Recent advances in carotenoid absorption, distribution, and elimination^☆^

**DOI:** 10.1016/j.bbalip.2025.159619

**Published:** 2025-04-28

**Authors:** Wafa’a Hajeer, Amparo Blanco, Anthony P. Miller, Jaume Amengual

**Affiliations:** a Division of Nutritional Sciences, University of Illinois at Urbana-Champaign, Urbana, IL, USA; b Department of Food Science and Human Nutrition, University of Illinois at Urbana-Champaign, Urbana, IL, USA

**Keywords:** β-carotene oxygenase, Xanthophylls, Carotenes

## Abstract

Carotenoids are a class of pigments with antioxidant properties synthesized by photosynthetic and heterotrophic organisms. Humans can store carotenoids in their intact form or cleave them enzymatically to apocarotenoids such as vitamin A, a hormone-like nutrient with crucial roles in gene expression and vision. Clinical and preclinical studies suggest that the consumption of diets rich in carotenoids attenuate cardiometabolic diseases, some types of cancer, neurodegenerative disorders, and inflammatory conditions. The bioactive properties of carotenoids depend, at least in part, on their accumulation in target tissues. However, the pathways that drive carotenoid absorption, delivery, and accumulation in tissues remain largely uncharacterized. This review provides a critical overview of the experimental models utilized to monitor carotenoid homeostasis in mammals. We also delve into recent findings concerning carotenoid intestinal uptake, bodily distribution, cellular uptake, and intracellular trafficking. Finally, we discuss the physiological relevance of a fecal carotenoid elimination pathway that operates independently of carotenoid enzymatic cleavage. Establishing the players governing carotenoid biodistribution and elimination is essential to maximize the bioactive properties of carotenoids in humans to prevent chronic diseases.

## 1. Introduction

Carotenoids are a group of terpenoids synthesized by plants, algae, bacteria, and fungi. Although some carotenoids such as phytoene and phytofluene are colorless, most carotenoids have vibrant colors ranging from yellow to red. Chemical modifications of carotenoids, such as the addition of oxygen groups, isomerization, or elongation, result in a wide diversity of configurations that confer these molecules unique biological properties. Carotenoids are classified as carotenes or xanthophylls based on the presence of oxygen: Carotenes are pure hydrocarbons, while xanthophylls contain oxygen in their structure, often as hydroxyl or other functional groups [1,2].

Carotenoids can also be classified based on their capacity to produce vitamin A in (1) provitamin A carotenoids, which have at least one unsubstituted β-ionone ring, and (2) non-provitamin A carotenoids. β,β’-carotene (β-carotene) is the most studied carotenoid, as it is the primary vitamin A precursor in the human diet and the only compound with two β-ionone rings. Other provitamin A carotenoids in the human diet are α,β-carotene (α-carotene), and β-cryptoxanthin, which contain a single β-ionone ring. Provitamin A carotenoids account for approximately 70 % of vitamin A in people following an omnivore diet and are the only vitamin A source for strict vegetarians [3,4]. Other carotenoids commonly found in our diet and tissues are lycopene, lutein, and zeaxanthin; however, these compounds lack provitamin A activity [5,6].

In photosynthetic organisms, carotenoids serve a dual role during photosynthesis, acting as accessory pigments to maximize light absorption, and participating in the dissipation of the excess of energy from chlorophylls under intense sunlight conditions. Plants express several carotenoid-cleaving enzymes to generate many different apocarotenoids. Some of these derivatives are the precursors of phytohormones such as abscisic acid and strigolactones, which participate in the development and growth of the plant [7,8], in a comparable fashion to vitamin A’s role in cell growth and differentiation in mammalian tissues. Fungi lack photosynthesis; however, carotenoids play key roles in these organisms. For example, fungal apocarotenoids have signaling properties in reproduction [9] or fungal infection [10]. Some fungi can also produce retinal, the first vitamin A intermediate and the visual chromophore in mammals. As it occurs in the human eye, retinal produced in fungi binds to opsins to regulate responses to light [11]. For a recent overview of fungal carotenoid occurrence and synthetic pathways see [12–15].

Consumption of carotenoid-rich foods, and their presence in our tissues, are positively associated with improved health outcomes such as reduced risk of cardiometabolic diseases, cancer, and cognitive disorders [16–21]. For example, the macula lutea accumulates lutein and zeaxanthin, two xanthophylls typically found in green leafy vegetables and egg yolk. Studies show that macular carotenoids have a protective effect against the development of age-related macular degeneration by protecting the retina from the excess of UV light [22,23]. Also, consuming lycopene-rich foods such as tomato has been associated with prostate cancer prevention, as lycopene is present in prostate tissue, which could influence tumor development either as an intact form or apocarotenoid derivatives [24–26]. Lastly, clinical data support that the accumulation of carotenoids in the skin could protect against erythema (sunburn) [27,28].

This review summarizes the recent findings on carotenoid biodistribution, providing a critical overview of the experimental methodology utilized in the carotenoid field. It also highlights a novel elimination pathway, independent of carotenoid cleavage, recently identified in our lab.

## 2. Experimental models to study carotenoid absorption and biodistribution

### 2.1. Cell culture models

Because they lack multicellular complexity, cell culture models are typically used to study the properties of compounds, rather than their biodistribution pathways. However, some cell lines such as the human colon carcinoma Caco-2 cells can be used to evaluate intestinal carotenoid uptake. These models pose some limitations, for example, Caco-2 monocultures do not produce mucus, a gel-like substance produced by intestinal goblet cells that serve as a protective barrier and lubricant [29]. Some studies overcome this limitation by establishing co-cultures of Caco-2 cells with goblet cell-like models such as the HT29-MTX cells [30]. Results derived from these studies suggest that the presence of mucus facilitates carotenoid uptake [31], although these results were not consistent between groups [32].

A second limitation in the study of carotenoid absorption in cell cultures is the absence of standardized protocols for delivering carotenoids in cell culture media. The diverse chemical structure of carotenes and xanthophylls determines their polarity and solubility to common solvents. One can overcome this limitation by using potent detergents to create micelles to deliver carotenoids to cells by creating small pores in the plasma membrane, which can alter cellular integrity [33,34]. Milder approaches such as cyclodextrin, purified lipoproteins, or unilaminar vesicles enriched with carotenoids have been used [35–39], however, the stability and concentration of carotenoids reaching the cell might be limited. More importantly, it remains unclear which of these methods, if any, preserves the intracellular compartmentalization of carotenoids among organelles when compared to animals fed carotenoids. In another words, to study the pathways responsible for carotenoid uptake and intracellular distribution is imperative to ensure that the delivery methods do not interfere with the subcellular distribution of these compounds. A last potential caveat, especially when using immortalized cell lines, is the expression of adequate membrane receptors, transporters, and carotenoid-cleaving enzymes, as these proteins directly influence the degree of carotenoid accumulation. Novel technologies such as tissue organoids or gut-on-chips could be useful to evaluate carotenoid absorption in the intestine by achieving physiologically relevant conditions, and could contribute to reducing the need for *in vivo* studies [40].

### 2.2. Mice deficient in β-carotene oxygenase 1 (Bco1^−/−^) and 2 (Bco2^−/−^)

**Fig. 1.**
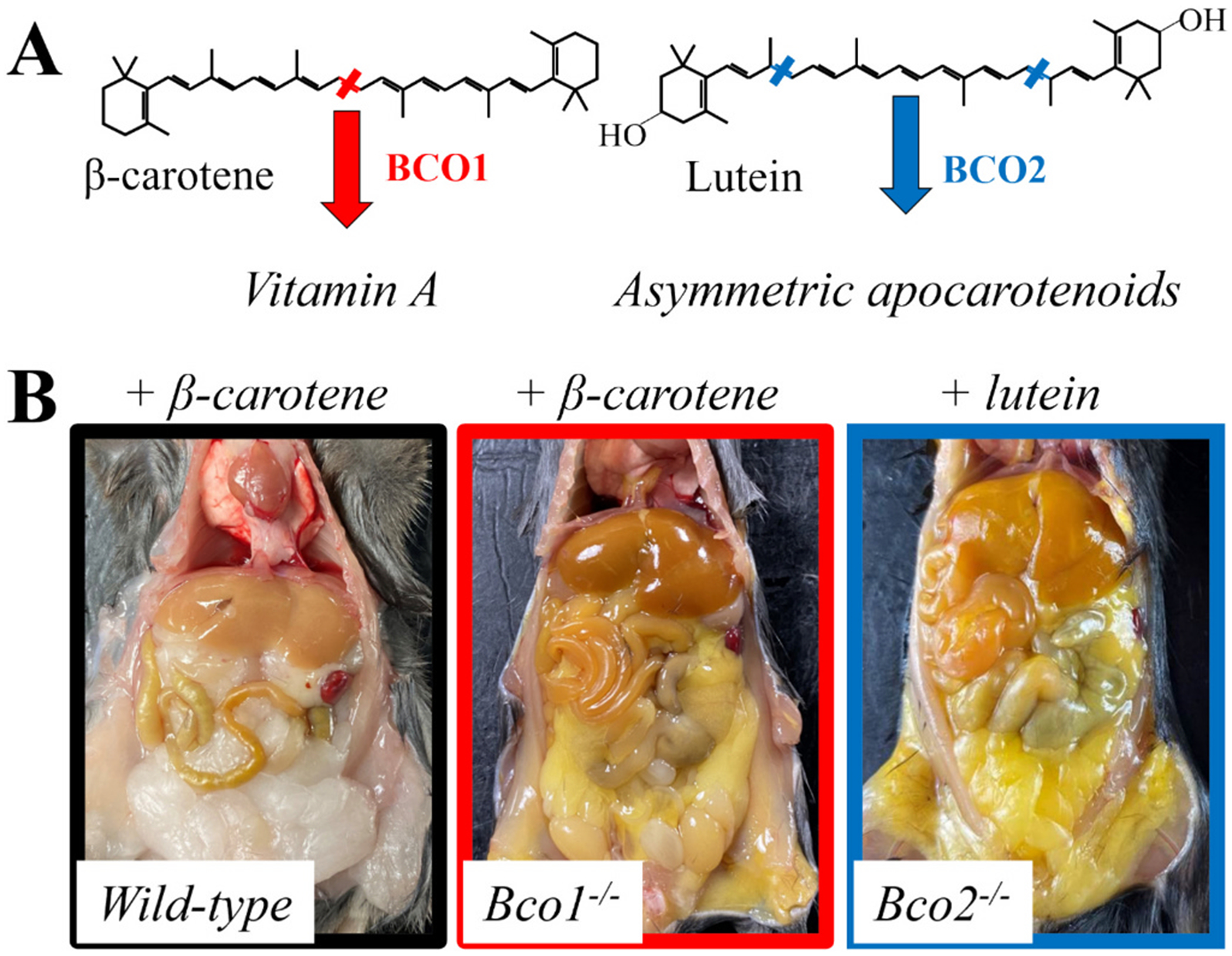
**(A)** The β-carotene oxygenase 1 (BCO1) and BCO2 are the only two enzymes capable of cleaving carotenoids in mammals to form symmetric (vitamin A) and asymmetric apocarotenoids. **(B)** Wild-type and *Bco1*^−/−^ mice were fed 50 mg/kg of β-carotene for four weeks. *Bco2*^−/−^ mice were fed 50 mg/kg of lutein for 4 weeks.

While humans accumulate significant amounts of carotenoids in plasma and tissues, wild-type mice do not. This phenomenon was initially attributed to differences in carotenoid absorption between humans and mice [41], however, evidences from our lab, and others, indicate that the relative distribution, expression levels, and enzymatic activity of the carotenoid-cleaving enzymes might be the main contributor to the interspecific differences observed between mice and human [42–47]. This experimental hurdle was overcome with the development of mouse models lacking the two carotenoid-cleaving enzymes in mammals: BCO1 and BCO2. At a glance, *Bco1^−/−^* mice fed β-carotene accumulate this compound in plasma and tissues, and when fed a diet containing β-carotene as the sole source of vitamin A, *Bco1^−/−^* mice can develop signs of vitamin A deficiency [45]. *Bco2^−/−^* mice accumulate xanthophylls such as lutein, zeaxanthin, and astaxanthin [44,48] (Fig. 1). Despite these studies, some researchers still utilize wild-type mice to carry out absorption and biodistribution studies by either downplaying or ignoring the significant differences between mice and humans.

Since their characterization, *Bco1^−/−^* and *Bco2^−/−^* mice have undoubtedly advanced the carotenoid field, contributing to the identification of intracellular carotenoid-binding proteins [37,38,49], intestinal transporters [50–52], lipoproteins responsible for carotenoid delivery to tissues [53,54], establishing the role of carotenoids in photoprotection and vision [42,55,56], and the antioxidant properties of these compounds in their intact form [57,58].

*Bco1^−/−^* and *Bco2^−/−^* mice have also been extremely valuable to dissect the effects of intact carotenoids and their apocarotenoid derivatives, including vitamin A, in various organs such as the adipose tissue, liver, lungs, and the eye [42,44,59–63]. For example, our group examines the effects of β-carotene in cardiometabolic diseases such as obesity, hyperlipidemia, and atherosclerosis. By comparing the differential effects of β-carotene supplementation in wild-type and *Bco1^−/−^* mice, our studies demonstrate that vitamin A formation drives the beneficial effects of β-carotene in lipid metabolism and inflammation (we recently reviewed this topic in the context of atherosclerosis in [16]).

Dietary interventions in *Bco1^−/−^* and *Bco2^−/−^* mice have been an essential tool for validating BCO1 and BCO2 enzymatic activity assays *in vivo* [64–67]. Comparative feeding studies utilizing wild-type, *Bco1^−/−^*, *Bco2^−/−^*, and *Bco1^−/−^Bco2^−/−^* mice reveal, for example, that BCO2 is the main responsible for cleavage of lycopene [68] and β-cryptoxanthin [43], but plays a minimal role the cleavage of β-carotene in mice [43]. Similarly, recent studies combining enzymatic assays with mouse studies revealed that BCO1 is the main responsible for the cleavage of neurosporaxanthin, a fungal carotenoid with provitamin A properties [64].

As highlighted above, wild-type mice are extremely efficient at cleaving carotenoids, therefore, studies aiming to examine the absorption and bioavailability of carotenoids using wild-type mice should be carefully interpreted [41]. For example, a recent study compared the bioavailability of four carotenoids by measuring plasma and intestinal levels after a single gavage, concluding that one of them, phytofluene, is more bioavailable than its counterparts (lycopene, β-carotene, and phytoene). However, the specific affinity of BCO1 and BCO2 for these compounds was not considered, nor the authors collected feces to quantify the differential absorption of these molecules [69]. The limited accumulation of carotenoids achieved in wild-type mice can be overcome by administering supraphysiological doses, either through feeding or intraperitoneal injection. While these methods can temporarily increase carotenoids levels in tissues, they are not recommended if the study aims to investigate the physiological routes dictating carotenoid biodistribution.

### 2.3. Unconventional animal models in carotenoid research

Animals such as ferrets (*Mustela putorius* ssp. *furo*) have been extensively utilized to model carotenoid uptake, storage, and distribution for over two decades, (extensively reviewed in [70]). Other models such as preterm piglets have recently gained interest modeling carotenoid metabolism [71,72], opening new avenues in the study of carotenoids intestinal absorption. Similarly, studies in various non-human primates have provided valuable insights into biodistribution pathways, retinoid production, and accumulation in the brain, where these compounds could contribute to improving cognitive functions [73–77].

A significant limitation of these models in comparison to mice lies on their lack of versatility, particularly in the availability of knockout models for genetic studies. Additionally, these models are more costly to maintain and typically raise greater ethical concerns, especially outside of the scientific community [78].

### 2.4. Methods to quantify carotenoid status and distribution pathways in humans

Carotenoids are readily detectable in plasma and tissues by high-pressure liquid chromatography (HPLC) using a ultraviolet-visible diode array detector. While this method offers high detection sensitivity, HPLC systems may not always distinguish closely related compounds. This limitation can be overcome utilizing more sophisticated methods such as mass spectrometry. Other approaches such as resonance Raman spectroscopy (RRS) can be utilized to assess skin carotenoid levels, and its combination with confocal microscopy, allows to distinguish the distribution of carotenoids at the cellular level [79–81]. Skin carotenoids measured by either RRS or reflection microscopy have their respective limitations, as these technologies remain expensive for widespread utilization [82].

Recently, the utilization of non-invasive devices such as the Veggie Meter using reflection spectroscopy to detect carotenoids in the human eye and skin has gained interest in monitoring carotenoid status in people [83]. These methods are relatively affordable; however, they also have limitations. For example, studies suggest that reflection microscopy do not efficiently detect skin lycopene levels [84], which accounts for one of the most abundant carotenoids in Western diets [85]. Other studies report that the Veggie Meter, which is gaining momentum in the nutrition field, also exhibits lower correlations than RRS with plasma carotenoids measured by HPLC, displaying higher intra and inter-device variability [86–88].

The use of Carbon-13 (^13^C) to label carotenoids allows the detection of trace levels of these compounds utilizing mass spectrometry. This approach allows to track the distribution of carotenoids in humans and monitor their clearance in the blood, isomerization, and in cases such as ^13^C-β-carotene, their conversion to ^13^C-vitamin A derivatives [89–91]. These studies revealed that ^13^C-carotenoids remain in circulation for weeks after single dose, but the fate of these compounds in tissues, or how long it requires until their complete clearance from the body remains unclear. A major limitation in these studies is the limited availability of commercially sourced ^13^C-carotenoids. Due to this, some laboratories opted to generate ^13^C-carotenoids utilizing ^13^C-glucose [74,92].

## 3. Factors affecting carotenoid absorption in the intestine

### 3.1. Influence of the food matrix in carotenoid uptake

Once ingested, carotenoids are incorporated into mixed micelles containing other lipids such as bile acids, phospholipids, and triglycerides that enable intestinal absorption by the enterocyte [93]. As it occurs with other lipophilic molecules, carotenoid absorption increases in the presence of dietary fat.

The chemical form in which carotenoids are present in the food matrix also can affect intestinal absorption: For example, lycopene forms crystalline aggregates that can limit its absorption, while the esterification of lutein and zeaxanthin promotes it [94,95]. For a recent review of the role the food matrix plays in carotenoid absorption see [96].

### 3.2. Carotenoid polarity

Interventional studies in human suggest intestinal carotenoid absorption and bioavailability are directly associated with their polarity [97–101], however, intrinsic genetic factors, which are known to affect carotenoid metabolism, are not typically taken into account. To overcome these limitations, we recently compared the effect of carotenoid polarity in *Bco1^−/−^Bco2^−/−^* mice fed a vitamin A deficient diet, which favors intestinal carotenoid uptake (see below).

By using sex-matched *Bco1^−/−^Bco2^−/−^* littermates, we could evaluate different carotenoid species without the interference of any carotenoid-cleaving enzyme. After two weeks on a carotenoid-free, vitamin A deficient diet, we gavaged the mice once with β-carotene (no oxygen), β-cryptoxanthin (one hydroxyl group), and neurosporaxanthin (one carboxylic group). We collected fecal samples every 12 h for three consecutive days, and quantified carotenoid levels in feces, plasma, and liver to estimate the biodistribution and elimination of these compounds. Mice fed β-carotene disposed larger amounts of this compound in their feces in comparison to those fed β-cryptoxanthin and neuro-sporaxanthin. These mice also accumulated less β-carotene in plasma and liver than those fed xanthophylls [64]. Overall, our confirmed clinical data indicating that polarity is a major contributor to carotenoid intestinal uptake, validating the use of carotenoid-cleaving enzymes as “humanized” mouse models for carotenoid absorption.

A limitation of our study, however, is that neurosporaxanthin (C35) is smaller than β-carotene and β-cryptoxanthin, which contain both 40 carbon atoms each. Hence, we cannot exclude that part of the differences in intestinal absorption between groups are mediated by the size of these compounds. Additionally, because the isolation and purification of sufficient amounts of neurosporaxanthin to perform large-scale studies are extremely challenging processes, we only utilized three animals per group, which likely limited our statistical power [64].

### 3.3. Intestinal vitamin A status and scavenger receptor class B type 1 (SR-B1)

In 2008, Seino and colleagues identified the intestinal-specific homeobox (ISX) as a key regulator of vitamin A homeostasis in mammals. By comparing the expression of intestinal samples in *Isx^−/−^* to wild-type mice, they identified SR-BI and BCO1 as two key target genes for ISX in the enterocyte [102]. Follow-up studies by the von Lintig’s lab identified ISX as a retinoic acid-sensitive transcription factor that directly binds the promoter region in the genes encoding BCO1 and SR-B1 [50,51]. In the presence of vitamin A, the upregulation of ISX blocks SR-BI and BCO1 expression. By attenuating the expression of SR-BI and BCO1, ISX limits carotenoid uptake and hinders vitamin A, respectively. Once vitamin A is in the enterocyte, it is esterified by the lecithin: retinol acyltransferase (LRAT) and then packed into chylomicrons for release into the lymphatic system. In the absence of LRAT, the flux of vitamin A towards retinoic acid dramatically enhances ISX expression in the enterocyte [103], highlighting the contribution of retinoid esterification in the regulation of vitamin A homeostasis [104,105].

Recently, Quadro’s group demonstrated that ISX is also expressed in the placenta, identifying this transcription factor as a mediator of vitamin A homeostasis beyond the intestine [106]. As it occurs in the enterocyte, placental ISX is upregulated by retinoic acid, which in turn, regulates BCO1 and SR-B1 expression in this organ. Placental ISX participates in the regulation of the microsomal triglyceride transfer protein (MTP), which in hepatocytes and enterocytes contributes to the lipidation of newly synthesized lipoproteins [107]. The authors reported a biphasic effect in which β-carotene supplementation and vitamin A production first upregulate ISX expression to decrease MTP levels, followed by a decrease in ISX expression likely due to the asymmetric β-carotene cleavage to produce apo-10′-carotenal. These data suggest that asymmetric apocarotenoids affect placental lipoprotein lipidation, which constitute a major energy supply for the developing embryo [108]. These exciting findings warrant further investigations, and align with results in mice and cell culture demonstrating that vitamin A reduces hepatic lipoprotein lipidation [109].

Follow-up studies by von Lintig’s group also identified SR-BI’s role as intestinal transporter of xanthophylls by comparing the levels of zeaxanthin in *Bco2^−/−^* and *Bco2^−/−^Scarb1^−/−^* mice fed this carotenoid [50]. The absence of ISX not only increased zeaxanthin levels, but it also resulted in the accumulation of vitamin E in the liver [50]. Our lab expanded these findings by manipulating ISX expression using fenretinide, a synthetic retinoid utilized in cancer therapy [110]. Fenretinide downregulated SR-B1 in the gut, limiting the uptake of β-carotene, lutein, and vitamin E in plasma and tissues [111]. In agreement with these findings, fecal carotenoids and vitamin E in fenretinide-treated mice increased in comparison to vehicle-treated mice [111], overall highlighting the importance of vitamin A signaling in carotenoid and vitamin E homeostasis.

### 3.4. Cluster of differentiation 36 (CD36)

Before the identification of SR-B1 and other intestinal lipid transporters, the uptake of carotenoids in the enterocyte was believed to depend on diffusion across the plasma membrane [112]. In 2002, von Lintig’s group identified the gene product of the NinaD gene as a class B scavenger receptor in *Drosophila*. NinaD mutant flies displayed a blind phenotype due to the lack of carotenoids in the eye, which led to the identification of NinaD as a carotenoid transporter. The protein product of NinaD displays significant homology to its mammalian counterparts SR-BI and CD36 [113,114], which possess similar tridimensional structure, size, and ligand specificity [115]. SR-B1 and CD36 are expressed in similar cell types including the enterocyte, where they localize to the brush border [93]. Given the strong evidence that SR-B1 mediates carotenoid uptake (see above), it is also plausible that CD36 may play a role in this process.

Experimental data suggest that, indeed, CD36 participates in the uptake of carotenoids in the adipose tissue [116]. CD36 over-expression in human embryonic kidney and COS-1 cells promotes carotenoid uptake, while the inhibiting CD36 in these models leads to the opposite results [116,117]. In 2023, a study reported that CD36 is responsible for the uptake of astaxanthin [118], a xanthophyll found in seafood with potent antioxidant properties [119]. The authors utilized various experimental models including wild-type and *Cd36^−/−^* mice gavaged with a single dose of astaxanthin prior tissue harvesting. HPLC quantifications performed at different sections of the intestine suggested that CD36 is responsible for the uptake of astaxanthin [118]. However, the authors failed to acknowledge that wild-type mice do not accumulate significant amounts of xanthophylls in tissues when these compounds are provided in the diet at concentrations typically found in foods and vegetables [44]. Indeed, wild-type mice accumulate 10 times less astaxanthin than *Bco2^−/−^* mice [48].

Clinical data describe associations between *CD36* gene variants and carotenoid plasma levels [116,120,121], however, the impact of these variants on CD36’s function remains unclear. This knowledge gap prompted us to generate *Bco1^−/−^Cd36^−/−^* mice and compare them to *Bco1^−/−^* mice. To our surprise, the ablation of CD36 in mice increases β-carotene tissue levels (*Miller AP, unpublished*). These findings agree with those obtained in preclinical and clinical models by Abumrad’s group, where the depletion of CD36 increases gut permeability [122–124], suggesting that in the absence of CD36, dietary carotenoid are taken up at a faster rate. Studies using both *Bco1^−/−^Cd36^−/−^* and *Bco2^−/−^Cd36^−/−^* mice are needed to identify the role of CD36 in intestinal carotenoid uptake as well as in myeloid cells and adipocytes, where CD36 is highly expressed [125].

### 3.5. Nieman-Pick C1-like 1 (NPC1L1)

NPC1L1 was identified in 2004 as a critical mediator of intestinal cholesterol absorption [126], and its role in vitamin E uptake was identified shortly after in Caco-2 cells and rats [127]. While some studies indicate that NPC1L1 does not participate in the intestinal uptake of carotenoids [128,129], others suggest it could favor the uptake of some xanthophylls [130,131]. As is common in bioavailability studies, these experiments were conducted in cell culture or mice that express carotenoid-cleaving enzymes. In 2025, the von Lintig’s lab used the NPC1L1 inhibitor ezetimibe in *Bco1^−/−^Bco2^−/−^* mice to study the role of this transporter on intestinal carotenoid uptake [132]. HPLC results showed that NPC1L1 participates in the uptake of zeaxanthin, but not β-carotene [132], suggesting that NPC1L1 is selective for xanthophylls. Whether this phenomenon also occurs for other transporters will require further experiments.

### 3.6. Intestinal malabsorption

Some genetic conditions and metabolic surgeries result in nutrient malabsorption, which typically affect lipophilic compounds, including carotenoids. For example, a recent study by Harari and colleagues compared blood carotenoid levels in individuals at baseline and at six months after undergoing bariatric surgery [133]. HPLC quantifications revealed a marked decline in plasma carotenoids of over 65 % in post-surgery samples, in agreement with similar studies [134]. Comparable outcomes are described in subjects expressing defective forms of *MTP*, which in the gut participates in the lipidation of chylomicrons [135]. Under both circumstances, recommendations to consume high doses of lipophilic vitamins such as vitamin A and E exist to curb the development of deficiencies in these populations. However, there are no specific guidelines aiming to prevent hypocarotenemia [136,137], a condition that could hinder the bioactive properties of carotenoids in tissues such as the eye.

A study published in 2024 highlighted the effect of bariatric surgery on key genes implicated in vitamin A intestinal homeostasis. Gene expression in gastrointestinal mucosa collected from 20 women before and three months after surgery revealed an increase in vitamin A signaling after surgery in different portions of the intestine [138]. Because participants received daily vitamin A supplements, it is not possible to determine whether changes in gene expression occurred because of bariatric surgery *per se* or because of the effect of vitamin A dosing on gene regulation. Interestingly, gene expression results in the duodenum were comparable to those reported in the ileum and jejunum, despite the bolus of food did not travel *via* this segment after surgery. Changes on gene expression could be attributed to the surgery itself or to the secretion of vitamin A metabolites to the common bile duct [139], which remains intact in metabolic surgeries.

## 4. Pathways responsible for carotenoid mobilization, storage, and intracellular transport

### 4.1. Enterocyte → blood

It is estimated that up to 40 % β-carotene in the enterocyte remains intact upon its uptake, while the remaining β-carotene would be converted to vitamin A by BCO1 [140,141]. Due to the minimal expression of BCO2 in the human intestine [142], it is assumed that most xanthophylls remain intact after intestinal absorption. After a meal, carotenoids and other dietary lipids are incorporated into nascent chylomicrons. As it occurs with other neutral lipids, MTP also may be the main responsible for the incorporation of carotenoids into nascent chylomicrons. This idea is based on Quadro’s recent studies unveiling the regulatory mechanisms of carotenoid delivery to the embryo, where they demonstrate that MTP transfers β-carotene to placental lipoproteins [106,143–145].

### 4.2. Blood → tissues

Once in circulation, chylomicrons release part of their lipid cargo by interacting with the endothelial cell-bound lipoprotein lipase (LPL) to become chylomicron remnants. Carotenoids within these remnants are thought to be taken up by hepatocytes through the low-density lipoprotein receptor (LDLR) and other membrane proteins with an affinity for apolipoprotein E (apoE) [146–148].

In humans, carotenoids are distributed among various lipoprotein species. This distribution can be predicted by their polarity: Xanthophylls such as lutein are enriched in high-density lipoproteins (HDL), while carotenes such as β-carotene appear in very low and low-density lipoproteins (V)LDL [149,150]. Unlike humans, *Bco1^−/−^* and *Bco2^−/−^* mice transport carotenoids exclusively in HDL, regardless of their polarity [151]. In 2024, we compared the distribution of β-carotene between lipoprotein fractions in *Bco1^−/−^* and *Bco1^−/−^* *Ldlr^−/−^* mice. Our lipoprotein separation system coupled to HPLC quantification revealed that the absence of the LDLR is sufficient to redistribute β-carotene between HDL and (V)LDL fractions: In *Bco1^−/−^* mice, 100 % of β-carotene was present in the HDL, while in *Bco1^−/−^Ldlr^−/−^* mice, between 50 and 70 % of β-carotene was in the (V)LDL fraction as it occurs in human plasma [152]. These results suggest that limiting lipoprotein clearance perturbs β-carotene among lipoprotein fractions. Further studies evaluating the distribution of other carotenoids in the absence of LDLR will be necessary to determine the contribution of this receptor and lipoprotein clearance to the partitioning of dietary carotenoids *in vivo* and its potential consequences to carotenoid distribution.

### 4.3. Hepatic and adipose tissue carotenoid reservoirs

As it occurs with other lipophilic compounds, carotenoids preferentially accumulate in the liver and adipose tissue in people [141,153–155], and within the cell, carotenoids are primarily stored in intracellular lipid droplets [151,156]. We recently observed that carotenoids stored in these organs can be mobilized [152] (see Section 5 for details). Genetic variants are associated with adipose tissue carotenoid levels, as reported by Borel’s group utilizing adipose tissue biopsies obtained in healthy men [157]. It is unclear whether these proteins, or others, also participate in the storage of hepatic carotenoids.

### 4.4. Low-density lipoprotein receptor (LDLR)

The LDLR, a cell membrane glycoprotein that mediates the endocytosis and degradation of apoB and apoE-containing particles, participates in one of the earliest and best-characterized aspects of lipoprotein metabolism [158]. The binding of (V)LDLs with LDLR results in endocytosis of the entire particle, followed by its delivery to the lysosome for degradation [159]. The LDLR is mostly expressed in the liver, where it mediates approximately 70 % of (V)LDL uptake [158,160]. LDLR is also expressed in a multitude of peripheral tissues, including the adipose tissue, lung, and the basolateral membrane of the enterocyte.

As it occurs with other aspects of mammalian physiology, including carotenoid metabolism (see above), murine models do not fully mimic some major aspects of human lipoprotein metabolism. For example, rodents secrete hepatic apoB48 and apoB100-VLDL, while humans only produce apoB100-VLDLs. Murine apoB48-(V)LDL are secreted and cleared at a greater rate than apoB100-(V)LDL, and also lack a binding site for the LDLR [161,162]. Because of this, in part, mice heavily rely on HDL for lipid transport, whereas humans primarily utilize the (V)LDL pathway.

Since their development [163], *Ldlr^−/−^* mice have vastly contributed to the study of atherogenesis, the main underlying cause of cardiovascular disease. In 2016, Quadro’s group was the first to describe the role of the LDLR in β-carotene hepatic uptake using *Ldlr^−/−^* mice. By injecting a single dose of β-carotene in the peritoneal cavity of *Ldlr^−/−^* and heterozygous *(Ldlr^+/−^*) mice, the authors observed that *Ldlr^+/−^* mice accumulated larger amounts of β-carotene in the liver than *Ldlr^−/−^* mice [148]. Despite the novelty of these findings, this study had several limitations: Intraperitoneal injections are not a physiological route for carotenoid delivery. The solubility of β-carotene in the intraperitoneal cavity, its diffusion towards the circulatory system, and its incorporation into lipoproteins could be dramatically affected by this unique administration route. Last, *Ldlr^−/−^* and *Ldlr^+/−^* mice express BCO1, and therefore, the accumulation of β-carotene in the hepatocyte could be limited by the formation of vitamin A [148].

In 2024, our lab examined the contribution of LDLR in carotenoid homeostasis by performing dietary studies in *Bco1^−/−^* and *Bco1^−/−^Ldlr^−/−^* mice fed β-carotene. *Bco1^−/−^* mice accumulated more β-carotene than *Bco1^−/−^Ldlr^−/−^* mice in the liver and adipose tissue, the two major organ carotenoid reservoirs. β-carotene levels in other organs such as the eyes and lungs remained unchanged between genotypes. Next, we utilized a liver-specific adeno-associated vector encoding LDLR to overexpress this receptor in *Bco1^−/−^* and *Bco2^−/−^* mice fed β-carotene and lutein, respectively. LDLR overexpression in hepatocytes stimulated carotenoid uptake in the liver at the expense of circulating and extrahepatic carotenoid pools [152]. These results confirm the contribution of LDLR in carotenoid tissue uptake and homeostasis.

### 4.5. Plasma lipid-transfer proteins

The phospholipid transfer protein (PLTP) and the cholesteryl ester transfer protein (CETP) constitute the two plasma lipid transfer proteins in humans [164]. PLTP is expressed in humans and mice and contributes to HDL maturation and size by facilitating the transfer of phospholipids between lipoprotein species [165]. CETP transfers cholesteryl esters from HDL to (V)LDL and triglyceride from (V)LDL to HDL [166,167], being a major contributor to the lipid composition in plasma lipoproteins. Mice do not express CETP, which could partially explain the dramatic differences in carotenoid distribution among lipoproteins between humans and mice. The role of CETP on carotenoid transfer has only been approached using *in vitro* models of isolated human and trout lipoproteins, although results derived from these studies failed to reach a consensus in the role of these enzymes in carotenoid transport [149,168].

### 4.6. HDL and HDL receptors

Over the past decades, HDL has gained popularity as a key mediator in cholesterol efflux from peripheral tissues, the first step in reverse cholesterol transport. However, HDL also plays a fundamental role in the delivery of cholesterol to some organs such as the adrenal gland [169]. Substantial evidence in clinical and preclinical studies indicates that HDL is a major carotenoid carrier to peripheral tissues, especially to the eye, as highlighted by Bernstein’s group using a variety of experimental approaches, including *Bco2*^−/−^ mice [53,54,170].

**Fig. 2.**
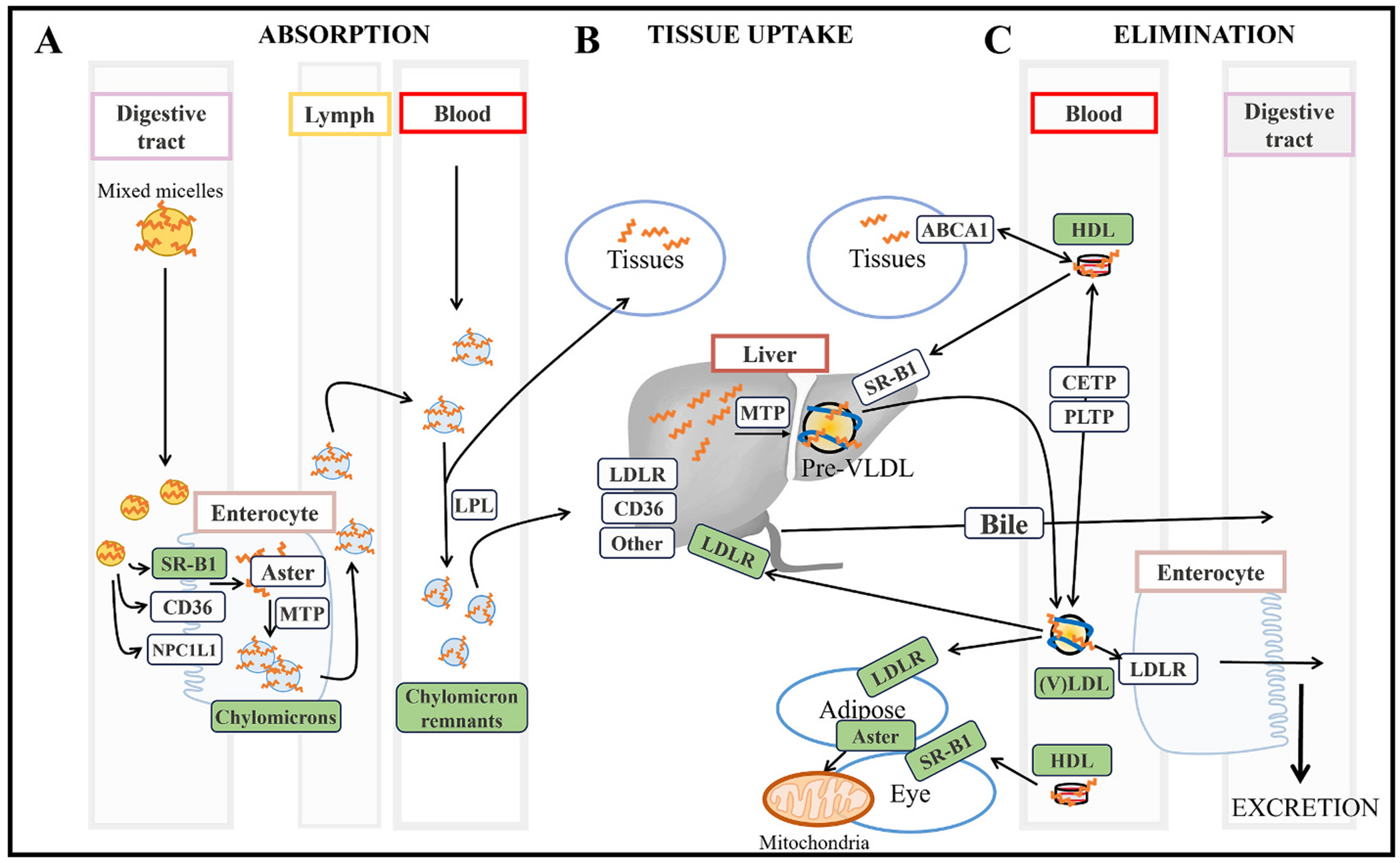
Overview of the molecular pathways and proteins involved in carotenoid **(A)** absorption, **(B)** tissue uptake, and **(C)** non-enzymatic elimination**.** Proteins highlighted in white represent those suspected of participating in carotenoid homeostasis, while proteins highlighted in green have been, in our opinion, sufficiently validated. SR-B1; Scavenger receptor class B member 1, CD36; cluster of differentiation 36, NPC1L1; Niemann-Pick C1-like 1, MTP; microsomal triglyceride transfer protein, LPL; lipoprotein lipase, LDLR; Low-density lipoprotein receptor, ABCA1; ATP-binding cassette transporter A1, VLDL; very low-density lipoprotein, HDL; high-density lipoprotein. (For interpretation of the references to colour in this figure legend, the reader is referred to the web version of this article.)

Genetic variants in HDL components such as apoAI, CETP, or leci-thin:cholesterol acyl transferase as well as in HDL receptors like SR-B1 or ATP-binding cassette transporter A1 (ABCA1) are associated with altered macular carotenoid concentrations [171–174]. Among the different HDL receptors, ABCA1 seems to play a major role in carotenoid homeostasis in ocular and extraocular tissues. For example, Wisconsin hypoalpha mutant chickens, which are characterized by HDL deficiency as a result of mutated ABCA1, display low levels of ocular carotenoids [175]. Similarly, patients afflicted with Tangier disease, which is characterized by loss-of-function variants for ABCA1, display characteristic orange tonsils [176], which served to quickly identify these patients in clinical practice. Altogether, experimental and clinical data suggest that ABCA1 could participate in the bidirectional transport of carotenoids (Fig. 2).

### 4.7. Aster proteins

The von Lintig’s group was the first to identify Aster-A and B as carotenoid-binding proteins [38]. Binding and modeling studies showed that Aster-A, B, and C bind xanthophylls, but not carotenes such as lycopene or β-carotene [38,132]. Upon binding, Aster proteins would mediate the trafficking of these lipids to the mitochondria, where xanthophylls typically accumulate in mice lacking at least one carotenoid-cleaving enzyme (For a recent review see [177]). Interestingly, we recently reported that lycopene preferentially accumulates in the mitochondria of mice, challenging that Aster proteins are the only mechanism by which carotenoids reach this organelle [68].

The initial characterization of Aster proteins back in 2018 by Tontonoz’s group demonstrated that these proteins mediate intracellular cholesterol transport acting downstream of the SR-BI membrane receptor [178]. In 2023, Tontonoz’s group also revealed that Aster-B and Aster-C also mediate the uptake of dietary cholesterol in the enterocyte, acting downstream to the cholesterol acceptor NPC1L1, to deliver cholesterol from the brush membrane to the endoplasmic reticulum [179]. In 2025, the von Lintig showed that Aster-C directs zeaxanthin to the mitochondria in enterocytes [132]. Again, this transport route is not shared by β-carotene, which would directly interact with BCO1 to form vitamin A in the cytosol [132]. Studies testing the role of Aster proteins, as well as other carotenoid-binding proteins [180], in the intracellular mobilization of each major dietary carotenoid will further consolidate the role of these transporters in human physiology.

## 5. Non-enzymatic carotenoid elimination

Mammals, including humans, lack the adequate enzymes to degrade the cholestane ring present in cholesterol. Therefore, we must dispose of this compound *via* the fecal route to prevent the excessive accumulation of this lipid in tissues [181]. Approximately 30 % of cholesterol in humans is eliminated through the transintestinal cholesterol elimination (TICE) pathway, while the rest is excreted *via* the biliary route [182,183]. The TICE pathway consists in the elimination of cholesterol in plasma lipoproteins *via* the reverse transport across the enterocyte.

LDLR in the basolateral membrane of the enterocyte is among the transporters implicated in TICE [182,183]. Therefore, we explored the role of this receptor in the elimination of β-carotene in *Bco1*^−/−^ and *Bco1*^−/−^ *Ldlr*^−/−^ mice fed β-carotene that were subsequently deprived of β-carotene for four days [152]. In the absence of LDLR, mice eliminated less β-carotene in their feces, implicating this receptor in a previously uncharacterized pathway for carotenoid homeostasis in mice [152].

These exciting results prompted us to explore whether carotenoids already stored in tissues are susceptible to being mobilized and eliminated by the feces. To this end, we utilized *Bco1*^−/−^*Bco2*^−/−^ mice fed β-carotene to completely rule out β-carotene cleavage by BCO2 in tissues [43,60]. After feeding mice for 12 weeks, we sacrificed a subset of mice as Baseline while the remaining mice continued on a carotenoid-free diet for two weeks (Washout). β-carotene levels in plasma and liver in the Washout group were dramatically reduced in comparison to Baseline mice. Importantly, β-carotene was detectable in the feces by HPLC two weeks after the diet switch to a carotenoid-free diet [152]. β-carotene levels in adipose tissue were also depleted in the Washout group to a lesser degree than plasma and hepatic stores, suggesting the presence of different mobilization pathways in different organs.

A plausible route for carotenoid disposal would be the biliary route, however, only two studies in the literature report the quantification of carotenoids in the bile. First, only a single report described the presence of carotenoids in human bile, describing a positive correlation between bile and plasma carotenoid levels [184]. A second report in ferrets, Wang’s group cannulated the lymphatic system to collect bile for four hours after dosing the animals with β-carotene, however, they did not detect β-carotene or its apocarotenoid derivatives [185]. Therefore, we quantified plasma and biliary β-carotene levels in *Bco1*^−/−^ mice fed this compound. Our HPLC quantifications showed a direct correlation between both values [152], as reported in humans [184]. These results further cemented the adequacy of *Bco1*^−/−^ mice to study β-carotene biodistribution. However, our study failed to distinguish between the biliary and non-biliary (transintestinal) routes.

## 6. Conclusions and future directions

The interest of carotenoids in biomedical research has steadily increased over the past decades, however, critical gaps in the pathways responsible for their accumulation in target tissues remain (Fig. 2). Yet, deepening our understanding of the role carotenoids play in health and disease will pave the way for innovative applications and therapeutic strategies focused on delivering these compounds to target organs and tissues, where they will carry out their bioactive properties. To this end, the selection of adequate experimental models that closely mimic humans is imperative. Establishing “humanized” mammalian models will contribute to reaching meaningful data, but prioritizing rigor in the carotenoid field is necessary. Even when using adequate mammalian models, one should also attempt to mimic the physiological carotenoid levels reported in people. Failing to do so could result in unpredictable biodistribution or elimination pathways that could lead to data misinterpretation.

## Data Availability

No data was used for the research described in the article.
